# CXCL16/CXCR6 axis arises as a potential peripheral biomarker of early COPD development – results from a pilot study

**DOI:** 10.3389/fmed.2025.1636360

**Published:** 2025-07-09

**Authors:** Patrice Marques, Irene Bocigas, Elena Domingo, Vera Francisco, Julia Tarrasó, Laura Piqueras, Jaime Signes-Costa, Cruz González, Maria-Jesus Sanz

**Affiliations:** ^1^Department of Pharmacology, Faculty of Medicine and Odontology, University of Valencia, Valencia, Spain; ^2^Institute of Health Research INCLIVA, University Clinic Hospital of Valencia, Valencia, Spain; ^3^CIBEREHD-Spanish Biomedical Research Centre in Hepatic and Digestive Diseases, Carlos III Health Institute (ISCIII), Madrid, Spain; ^4^Pneumology Unit, University Clinic Hospital of Valencia, Valencia, Spain; ^5^CIBERDEM-Spanish Biomedical Research Centre in Diabetes and Associated Metabolic Disorders, Carlos III Health Institute (ISCIII), Madrid, Spain

**Keywords:** cigarette smoking, COPD, GOLD 1, CXCL16, CXCR6, biomarker, inflammation

## Abstract

**Background:**

Chronic obstructive pulmonary disease (COPD) is mainly caused by long-term exposure to cigarette smoke. Since systemic inflammation is an important component of COPD pathophysiology, its characterization is essential for developing new biomarkers and pharmacological approaches. We have previously reported CXCL16/CXCR6 axis upregulation, a key element of leukocyte trafficking in COPD. Given the paucity of data on early-stage COPD patients (GOLD 1), we investigated CXCL16/CXCR6 axis expression in this population and in individuals at risk for developing COPD.

**Design:**

Blood samples were collected from 27 GOLD 1 patients, 27 symptomatic smokers with normal lung function (pre-COPD), and 14 non-smokers. CXCR6 expression was assessed in platelets, leukocytes, and leukocyte-platelet aggregates by flow cytometry. Plasma CXCL16 levels were measured by ELISA and lung function by spirometry.

**Results:**

CXCL16 plasma levels and CXCR6 expression on platelets, classical monocytes, B-cells, and leukocyte-platelet aggregates were higher in GOLD 1 patients than in non-smokers and pre-COPD subjects. While CXCR6 expression was similar between the pre-COPD group and non-smokers, plasma levels of CXCL16 were higher in the former. Finally, CXCL16/CXCR6 axis expression negatively correlated with FEV1/FVC ratio.

**Conclusion:**

This pilot study provides the first evidence that the CXCL16/CXCR6 axis is upregulated in early-COPD development. Increased CXCL16 plasma levels in GOLD 1 patients and pre-COPD subjects suggest CXCL16 as a potential peripheral biomarker of early COPD development. Given the importance of the CXCL16/CXCR6 axis in leukocyte trafficking, it may emerge as a druggable target to attenuate lung immune cell infiltration and prevent COPD development and progression.

## 1 Introduction

Chronic obstructive pulmonary disease (COPD) is a progressive lung disease characterized by persistent respiratory symptoms, including chronic bronchitis and emphysema, resulting in airflow limitation (1). It is primarily caused by long-term exposure to harmful particles or gases, with cigarette smoke being the most common risk factor. In accordance with the severity of COPD as defined by the Global Initiative for Chronic Obstructive Lung Disease (GOLD), patients are classified as having mild (GOLD 1), moderate (GOLD 2), severe (GOLD 3), or very severe (GOLD 4) (2).

Systemic inflammation has been described as an important component in the development and progression of COPD, and can lead to various comorbidities (1, 3). In addition, the enhanced adhesiveness of circulating leukocytes to the pulmonary endothelium leads to leukocyte trafficking into the lungs, which is key to establishing lung inflammation in COPD (4). However, there is a lack of data on systemic inflammation in patients with early-stage COPD (classified as GOLD 1) and in the population at risk for developing COPD. Indeed, most studies have collected data from patients with all types of COPD irrespective of disease severity (5–10). As a result, the percentage of GOLD 1 patients and subjects at risk of developing COPD in these studies is usually unknown or very limited.

Understanding the systemic inflammation associated with COPD is essential for the discovery of biomarkers and potential treatment options to improve both pulmonary and systemic health. In particular, pharmacological strategies to reduce leukocyte-endothelium interactions have the potential to translate into new treatments to prevent the development and progression of COPD.

CXCL16 is a chemokine expressed in two distinct forms: the transmembrane form (expressed on several cells including endothelial cells) promotes the firm adhesion of cells expressing its counter receptor CXCR6 (e.g., monocytes and lymphocytes), and the soluble form acts as a chemoattractant for CXCR6^+^ cells. Data from our previous study on the CXCL16/CXCR6 axis showed that CXCR6 expression on circulating leukocytes is enhanced in patients with COPD (11). This led to a partial increase in CXCR6-dependent leukocyte adhesion to the dysfunctional endothelium, which was significantly reduced by endothelial CXCL16 neutralization. However, because the few studies that have addressed the involvement of the CXCL16/CXCR6 axis in this pathology have included all types of COPD (11, 12), no firm conclusions have been drawn about its involvement in early stages of the disease. We recently reported that GOLD 1 patients show enhanced platelet reactivity, which seems to be one of the major triggers for the formation of leukocyte-platelet aggregates and subsequent leukocyte-platelet aggregate-endothelial adhesion (13).

Here, we hypothesized that the CXCL16/CXCR6 axis might also be initially upregulated in the early stages of COPD development. Therefore, we investigated CXCR6 expression on different immune players in patients with GOLD 1 and in subjects at risk of developing COPD, and explored its potential as an early diagnostic tool and/or strategy for therapeutic intervention.

## 2 Materials and methods

### 2.1 Human study population

Twenty-seven GOLD 1 patients, 27 long-term smokers without COPD (with normal lung function [LF]) and 14 non-smoker healthy volunteers were recruited from the Pneumology Unit of the University Clinic Hospital of Valencia (Valencia, Spain).

Fresh heparinized (17 IU/mL lithium heparin) and citrated (3.2% sodium citrate) blood samples (BD Vacutainer blood collection tubes, BD Biosciences, San Jose, CA) were collected, and pulmonary function tests were performed in all participants. To be eligible for the present study, the subjects had to meet all the inclusion criteria and none of the exclusion criteria, as detailed below:

*Inclusion criteria:*

• Diagnosis of mild COPD (GOLD 1): diagnosis based on clinical criteria with confirmation of irreversible obstruction on a functional test (spirometry) according to the GOLD 2023 guidelines (14) with a baseline post-bronchodilator forced expiratory volume in 1 second (FEV1)/forced vital capacity (FVC) < 0.7 and FEV1 > 80%.
• Long-term smokers with normal LF: Current or former smoking history of ≥ 10 pack-years with FEV1/FVC > 0.7, FEV1 > 80% and diffusing capacity of the lung for carbon monoxide (DLCO) > 80%.
• Non-smoker healthy volunteers with normal lung function: Non-smokers with FEV1/FVC > 0.7.

*Exclusion criteria:*

(1) Concomitant diagnosis of asthma; (2) history of inflammatory disease (rheumatoid arthritis, Crohn’s disease, etc.); and (3) use of anti-inflammatory drugs in the last 6 weeks.

FEV1 and FVC were determined by spirometry (MasterScreen PFT Body, Jaeger, Hoechberg Germany), while DLCO was quantified using an infrared analyzer (MasterScreen PFT Body, Jaeger), and then adjusted for hemoglobin values. All procedures were performed according to American Thoracic Society (ATS) and European Respiratory Society (ERS) guidelines (15).

The study complied with the principles outlined in the Declaration of Helsinki and was approved by the Institutional Ethics Committee of the University Clinic Hospital of Valencia (Ethical Approval Number: 2021/121).

Of note, patients in the GOLD 1 COPD group were individuals with an early diagnosis of COPD who were being followed up in the by the Pneumology Unit of the University Clinic Hospital of Valencia. The group of long-term smokers without COPD includes individuals which were being supervised by the same Pneumology Unit due to different respiratory symptoms associated to their smoking habit such as cough, sputum production, or dyspnea. Additionally, in this group were also included those being monitored for other conditions, such as obstructive sleep apnea syndrome (OSAS). Finally, healthy volunteers were hospital staff members or their relatives who met the inclusion criteria and had voluntarily undergone pulmonary function testing.

All participants were carefully selected to obtain age- and sex-matched groups, fully informed about the objectives and procedures of the study, invited to participate, and only those who voluntarily agreed signed a written informed consent form. The demographic and clinical features of participants are shown in Table 1.

**TABLE 1:** Demographic and clinical features of participants.

Features	A: Non-smoker volunteers (*N* = 14)	B: Normal LF smokers (*N* = 27)	C: GOLD 1 patients (*N* = 27)	*P-*value
Age (years)	57.71 ± 1.55	57.59 ± 1.16	60.78 ± 1.35	0.9982 (A *vs.* B) 0.3181 (A *vs.* C) 0.1668 (B *vs.* C)
Sex M/F (%)	6/8 (41.9/57.1)	21/6 (77.8/22.2)	14/13 (51.9/48.1)	0.0946 (A *vs.* B) > 0.9999 (A *vs.* C) 0.1601 (B *vs.* C)
Active smoking (%)	0 (0.0)	13 (48.1)	18 (66.6)	0.2709 (B *vs.* C)
CSE (packs-years)	0.00 ± 0.00	40.96 ± 4.68	39.89 ± 3.44	0.8199 (B *vs.* C)
DLCO (%)	N.D.	92.74 ± 2.38	70.56 ± 3.70††	**< 0.0001** (B *vs.* C)
FEV1/FVC ratio	77.91 ± 1.47	79.48 ± 0.94	65.07 ± 0.64**/††	0.3087 (A *vs.* B) **< 0.0001 (A *vs.* C)** **< 0.0001 (B *vs.* C)**
FEV1 (%)	106.80 ± 3.66	102.40 ± 2.90	93.26 ± 1.68**/†	0.8246 (A *vs.* B) **0.0085 (A *vs.* C)** **0.0382 (B *vs.* C)**

Data are presented as mean ± SEM. CSE, cigarette smoke exposure; DLCO, diffusing capacity of the lung for carbon monoxide; FEV1, forced expiratory volume in 1 second; FVC, forced vital capacity; GOLD, Global Initiative for Chronic Obstructive Lung Disease; LF, lung function; N.D., not determined. **P < 0.01 relative to non-smokers’ values. †*P* < 0.05 or ††*P* < 0.01 relative to normal LF smokers’ values. Values in bold denote statistical significance at the *P* < 0.05 level.

### 2.2 Soluble CXCL16 quantification

Plasma samples were obtained by centrifugation of heparinized human whole blood and stored at −80°C. Human plasma soluble CXCL16 was measured by an enzyme-linked immunosorbent assay (ELISA; DuoSet^®^ ELISA Kit, R&D Systems, Abingdon, United Kingdom–Catalog number: DY1164–Detection range: 15.6–1,000 pg/mL; sensitivity: 15.6 pg/mL; the specificity was evaluated by the manufacturer using several soluble factors tested at concentrations of 50 ng/mL, and none of these showed cross-reactivity or interference with the assay, supporting its high specificity for CXCL16). Both standards and samples were assayed in duplicate, and results are expressed as pg/mL of plasma soluble CXCL16.

### 2.3 Determination of CXCR6 expression on platelets and leukocyte subsets by flow cytometry

The expression of CXCR6 was determined on circulating platelets and different leukocyte subsets by flow cytometry. Full details, including the gating strategies (Supplementary Figures 1–6 and Supplementary Tables 1, 2) are described in the Supplementary material.

### 2.4 Statistical analysis

All results were analyzed using GraphPad Prism 6 (GraphPad Software, Inc., La Jolla, CA). Values are expressed as individual data points, percentages, or mean ± standard error of the mean (SEM), as appropriate. For comparisons of multiple groups, one-way analysis of variance followed by Tukey’s *post hoc* analysis was used for data that passed both the normality and equal variance tests; otherwise, the non-parametric Kruskal-Wallis test followed by Dunn’s *post hoc* analysis was used. In all analyses, *P*-values < 0.05 were considered statistically significant. In addition, some correlations between experimental findings and clinical characteristics were calculated using the Pearson and Spearman correlation tests.

## 3 Results

In total, 68 subjects were recruited and divided into 3 groups: GOLD 1 patients (*N* = 27), long-term smokers with respiratory symptoms and normal LF (pre-COPD; *N* = 27) and non-smoker controls (*N* = 14). Demographic and clinical characteristics of the participants are shown in Table 1. No significant differences were found between GOLD 1 patients and long-term smokers with normal LF with respect to age, sex, percentage of active smoking, or cumulative smoking exposure. As expected, the forced expiratory volume in 1 second (FEV1) and the FEV1/forced vital capacity (FVC) ratio were significantly lower in GOLD 1 patients than in the other groups.

### 3.1 Plasma CXCL16 levels are higher in normal LF smokers and GOLD 1 patients than in non-smoker controls, with GOLD 1 patients having the highest levels

**FIGURE 1:**
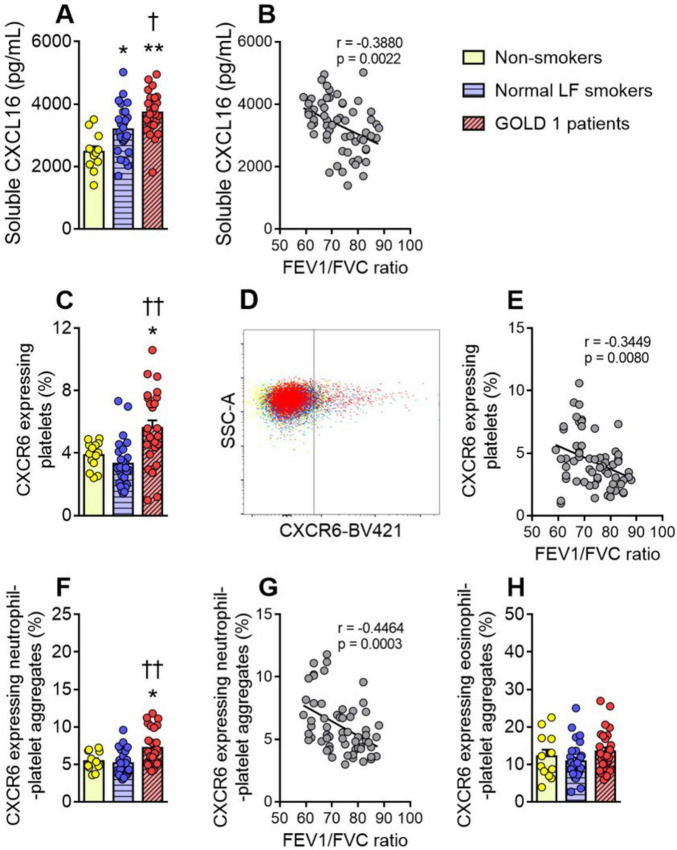
Enhanced circulating CXCL16 and CXCR6 expression on platelets and neutrophil-platelet aggregates in GOLD 1 patients. Plasma soluble CXCL16 levels (pg/mL) were measured by ELISA (**A**). Flow cytometry analysis of CXCR6-expressing platelets (**C**), and a representative dot plot (**D**). Flow cytometry analysis of CXCR6-expressing neutrophil-(CD16^+^CD41^+^) (**F**) or eosinophil-platelet aggregates (CD16^–^CD41^+^) (**H**). Results are presented as the percentage of positive (CXCR6^+^) platelets or leukocyte-platelet aggregates. Values are expressed as mean ± SEM. *P < 0.05 or **P < 0.01 relative to values in the respective non-smoker group. †*P* < 0.05 or ††*P* < 0.01 relative to values in the normal LF smoker group. Correlations between the FEV1/FVC ratio and the plasma levels of CXCL16 (**B**), the percentage of CXCR6-expressing platelets (**E**), and the percentage of CXCR6-expressing neutrophil-platelet aggregates (**G**). FEV1, forced expiratory volume in the first second; FVC, forced vital capacity.

A previous study by our group found that plasma levels of tumor necrosis factor-α (TNFα), an early indicator of inflammation, were higher in both GOLD 1 patients and normal LF smokers than in non-smoker controls of this cohort (13). As TNFα has been described to induce the expression and release of CXCL16 from endothelial and vascular cells (11, 16, 17), we first determined the circulating levels of this chemokine in the cohort. Plasma levels of soluble CXCL16 were significantly higher in GOLD 1 patients than in normal LF smokers and non-smoker controls (Figure 1A). Normal LF smokers also had higher circulating levels of this chemokine than non-smokers (Figure 1A). Of note, CXCL16 plasma levels correlated negatively with the FEV1/FVC ratio (Figure 1B), a key parameter of lung function in COPD.

### 3.2 CXCR6 expression in circulating platelets and neutrophil-platelet aggregates is higher in patients with GOLD 1 than in both normal LF smokers and non-smoker controls

We next evaluated the expression of the CXCL16 receptor, CXCR6, on platelets in this cohort. Flow cytometry analysis revealed that platelet CXCR6 expression was significantly higher in GOLD 1 patients than in the other two groups (Figures 1C, D), and no differences were observed between normal LF smokers and non-smoker controls (Figure 1C). Platelet CXCR6 expression negatively correlated with the FEV1/FVC ratio in these subjects (Figure 1E).

Similar results were found for neutrophil-platelet aggregates (Figures 1F, G), but not for eosinophil-platelet aggregates (Figure 1H). Because granulocytes do not express CXCR6 (11), no receptor expression was detected in platelet-free granulocytes (Supplementary Figures 7A, B).

### 3.3 CXCR6 expression in circulating monocytes is higher in GOLD 1 patients than in both normal LF smokers and non-smoker controls

**FIGURE 2:**
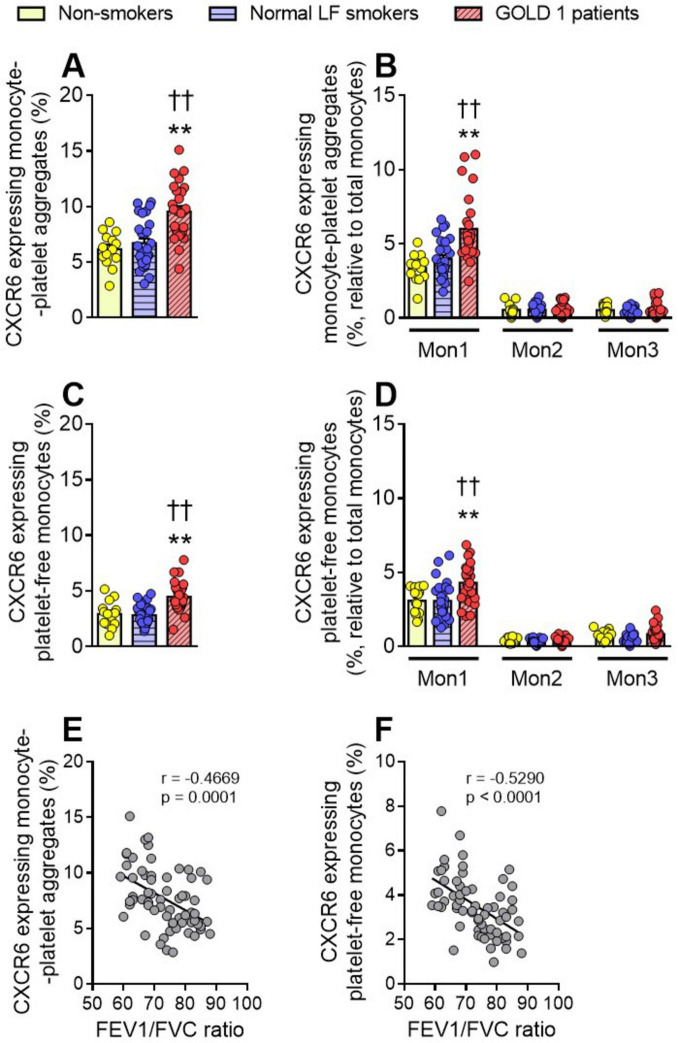
Increased CXCR6 expression was observed in monocytes and monocyte-platelet aggregates of GOLD 1 patients. Flow cytometry analysis of CXCR6-expressing monocyte-platelet aggregates (CD14^+^CD41^+^) (**A**) or platelet-free monocytes (CD14^+^CD41^–^) (**C**). The same analysis was done for the different monocyte subsets (Mon1: CD14^++^CD16^–^CCR2^+^; Mon2: CD14^++^CD16^+^CCR2^+^; Mon3, CD14^+^CD16^+^CCR2^–^), associated (**B**, CD41^+^) or not (**D**, CD41^–^) with platelets. Results are presented as the percentage of positive (CXCR6^+^) monocytes. Values are expressed as mean ± SEM. **P < 0.01 relative to values in the respective non-smoker group. ††*P* < 0.01 relative to values in the normal LF smoker group. Correlations between the FEV1/FVC ratio and the percentage of CXCR6-expressing monocyte-platelet aggregates (**E**), or the percentage of CXCR6-expressing platelet-free monocytes (**F**). FEV1, forced expiratory volume in the first second; FVC, forced vital capacity.

Monocytes can be classified into three subsets based on the differential expression of surface markers such as CD14, CD16, and CCR2 as classical, intermediate and non-classical monocytes (Supplementary Table 1). We measured CXCR6 expression on total monocytes and their subsets. We found that CXCR6 expression on monocyte-platelet aggregates (Figure 2A) and platelet-free monocytes (Figure 2C) was higher in GOLD 1 patients than in normal LF smokers and non-smoker controls, which was due to CXCR6 upregulation on the classical/Mon1 subset (Figures 2B, D). As expected, CXCR6 expression was higher in monocyte-platelet aggregates than in platelet-free monocytes in GOLD 1 patients (total monocytes: 9.54% *vs.* 4.47%, respectively). The percentage of CXCR6+ monocyte-platelet aggregates and CXCR6+ platelet-free monocytes also negatively correlated with the FEV1/FVC ratio (Figures 2E, F).

### 3.4 CXCR6 expression on circulating Th2- and Th17-lymphocyte-platelet aggregates is higher in patients with GOLD 1 than in both normal LF smokers and non-smoker controls

**FIGURE 3:**
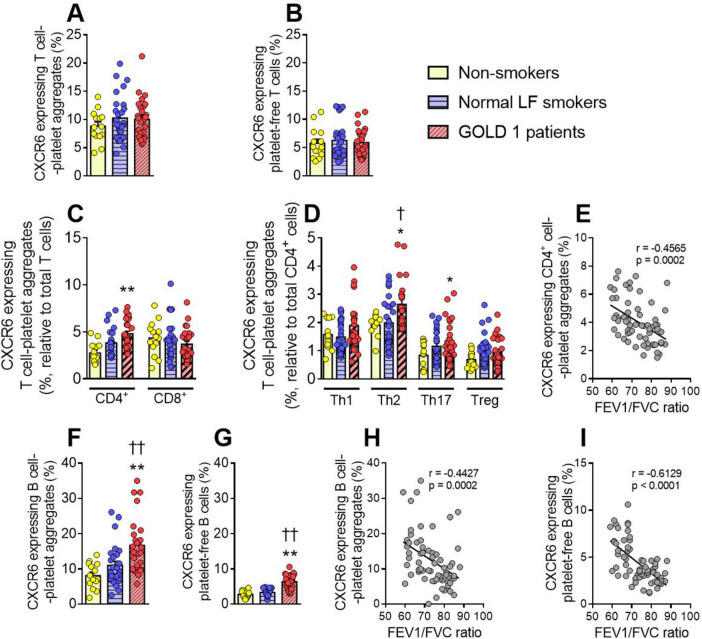
Augmented CXCR6 expression on B cells and several lymphocyte subset-platelet aggregates in GOLD 1 patients. Flow cytometry analysis of CXCR6-expressing T-cell-platelet aggregates (CD3^+^CD41^+^) (**A**), or platelet-free T-cells (CD3^+^CD41^–^) (**B**). The same analysis was done for CD4^+^ T-cell- and CD8^+^ T-cell-platelet aggregates (**C**), as well as for the different T-helper cell subsets (Th1: CXCR3^+^CCR6^–^; Th2: CXCR3^–^CCR6^–^; Th17: CXCR3^–^CCR6^+^) and regulatory T-cells (Treg: CD25^+^CD127^low^) aggregated to platelets (CD41^+^) (**D**). CXCR6 expression was determined by flow cytometry in B-cell-platelet aggregates (CD19^+^CD41^+^) (**F**) and platelet-free B-cells (CD19^+^CD41^–^) (**G**). Results are presented as the percentage of positive (CXCR6^+^) lymphocytes. Values are expressed as mean ± SEM. *P < 0.05 or **P < 0.01 relative to values in the respective non-smoker group. †*P* < 0.05 or ††*P* < 0.01 relative to values in the normal LF smoker group. Correlations between the FEV1/FVC ratio and the percentage of CXCR6-expressing CD4^+^ cell-platelet aggregates (**E**), B-cell-platelet aggregates (**H**), and platelet-free B cells (**I**). FEV1, forced expiratory volume in the first second; FVC, forced vital capacity.

We next evaluated the expression of CXCR6 on various T-lymphocyte subsets. No differences in CXCR6 expression were observed between the different groups in total T-lymphocyte-platelet aggregates (Figure 3A), platelet-free T-lymphocytes (Figure 3B), or CD8^+^ T-cell-platelet-aggregates and platelet-free CD8^+^ cells (Figure 3C and Supplementary Figure 7C). By contrast, CXCR6 expression on CD4^+^ T-cell-platelet aggregates was higher in GOLD 1 patients than in the other two groups (Figure 3C). A deeper analysis revealed that this effect seemed to be mainly due to T helper (Th)2 and Th17 subsets (Figure 3D). Again, the percentage of CXCR6 expressing CD4^+^ cell-platelet aggregates correlated negatively with the FEV1/FVC ratio (Figure 3E). In contrast to granulocytes, T-lymphocytes express CXCR6 (11, 18); however, the increased CXCR6 expression observed in these cells was entirely due to the contribution of platelets, as no differences were observed between groups in platelet-free CD4^+^ T-cells (Supplementary Figures 7C, D).

### 3.5 CXCR6 expression on circulating B-lymphocytes is higher in patients with GOLD 1 than in both normal LF smokers and non-smoker controls, and is partially due to the adhered platelets

Finally, we evaluated the expression of CXCR6 on circulating B-lymphocytes in this cohort and found that CXCR6 expression on both B-lymphocyte-platelet aggregates (Figure 3F) and platelet-free B-lymphocytes (Figure 3G) was higher in GOLD 1 patients than in normal LF smokers and non-smoker controls. Again, platelets seemed to partially contribute to the increase in CXCR6 expression, as expression was higher in B-lymphocyte-platelet aggregates than in platelet-free B-lymphocytes (16.70% *vs.* 6.29%, respectively). Similarly, we found negative correlations between CXCR6 expression on circulating B-lymphocytes and the FEV1/FVC ratio (Figures 3H, I).

## 4 Discussion

We provide evidence for an upregulation of the CXCL16/CXCR6 axis in early-stage COPD. Plasma soluble CXCL16 levels were significantly higher in both GOLD 1 patients and normal LF smokers than in non-smoker controls. Contrastingly, the expression of its counterreceptor, CXCR6, was elevated only on platelets, some leukocyte subset-platelet aggregates and some platelet-free leukocyte subsets in GOLD 1 patients, but not in the other two groups. Most of these altered parameters were negatively correlated with the FEV1/FVC ratio.

Few studies have determined the circulating levels of CXCL16 in the context of COPD. Indeed, only two reports have addressed this issue, and neither of them found differences between COPD patients and controls (11, 12). By contrast, we found here that both smokers with normal LF and GOLD 1 patients had higher plasma CXCL16 levels than non-smoker controls. Moreover, the plasma levels of CXCL16 were significantly higher in GOLD 1 patients than in smokers with normal LF. These discrepancies may due to differences in the recruited controls. For example, in one of these reports, ∼70% of the control subjects were active smokers (12), which probably influenced the basal circulating levels of the chemokine. In this regard, active smoking has previously been described to increase the levels of proinflammatory and prothrombotic mediators in plasma or serum (19–21). However, there are no data on circulating levels of CXCL16 beyond our results. Similarly, non-smoker subjects in the second report had several comorbidities: 41% had arterial hypertension (*vs.* 7% in the present study), 18% had type 2 diabetes (*vs.* 7% in the present study), and 29% had dyslipidemia (*vs.* 14% in the present study) (11). Again, these comorbidities might alter the basal levels of CXCL16. In support of this, cardiometabolic abnormalities and related complications have been associated with higher levels of CXCL16 (22, 23). Our finding that the plasma levels of CXCL16 were significantly higher in normal LF smokers (pre-COPD) and GOLD 1 patients than in control subjects suggests that circulating CXCL16 levels could be a new peripheral biomarker for COPD development in the susceptible population.

Regarding CXCR6 expression on different immune players, we show that CXCR6 was clearly upregulated in platelets from GOLD 1 patients. However, because of the increased platelet CXCR6 expression in these patients described here, together with the high platelet reactivity previously reported (13), upregulated CXCR6 expression was also found in neutrophil-, classical monocyte (Mon 1)-, Th2 cell-, Th17 cell-, and B-cell-platelet aggregates. In addition, among platelet-free leukocytes, CXCR6 upregulation was detected only in classical (Mon 1) monocytes and B-cells from GOLD 1 patients. Of note, most of the altered parameters presented here correlated negatively with the FEV1/FVC ratio, suggesting an association between the CXCL16/CXCR6 axis expression and initial airway obstruction. Although there is no universal consensus regarding *r* values and the strengths of the correlations, according to the *Statistics at Square One* from the British Medical Journal (BMJ) Publishing Group (24), we can assume that most of our correlations may be classified as moderate (*r* = 0.40–0.59) or strong (*r* = 0.6–0.79).

There is very little scientific evidence on CXCR6 in COPD. We previously demonstrated a significant increase in CXCR6 expression in platelets, neutrophil-, monocyte- and T-cell-platelet aggregates, as well as platelet-free monocytes and T-cells, in patients with COPD when compared with volunteers without COPD (11). However, few conclusions could be drawn from this study regarding CXCR6 expression in early-stage COPD, as only 8.7% of the recruited patients were classified as GOLD 1 (11). In addition to the present study, only one other report focused on CXCR6 expression in COPD (25), but specifically in lung CD8^+^ cells. In this study, increased CXCR6 expression in lung CD8^+^ T-cells was positively correlated with COPD severity; however, no differences were found between GOLD 1 patients and pre-COPD patients (25), which is consistent with our observations in peripheral blood CD8^+^ T-cells.

Notably, it has been demonstrated that the neutralization of endothelial CXCL16 significantly reduces leukocyte-platelet-endothelium interactions in COPD patients (11). Therefore, the upregulation of the CXCL16/CXCR6 axis described here in GOLD 1 patients is likely to be one of the contributors to the increased adhesiveness of leukocyte-platelet aggregates to the dysfunctional pulmonary endothelium described previously (13). Overall, it is tempting to speculate that anti-CXCL16/CXCR6 therapy in early-stage COPD may prevent disease progression and further complications.

Despite the novelty of these findings, we are aware that the limited sample size may compromise the generalization of the results. Moreover, there is a potential selection bias, as only patients without limitations in attending the hospital for pulmonary function testing were included, and the healthy volunteers consisted of hospital staff and their relatives, who may not represent the general population. In this sense, future research with larger and more diverse samples will be critical to validate these findings in the clinical practice. Nevertheless, we believe these findings might be a valuable contribution to future clinical research on COPD prediction, prognosis and treatment.

## 5 Conclusion

In conclusion, the present pilot study reports for the first time that the CXCL16/CXCR6 axis is upregulated in early-stage COPD. As a consequence, the increased plasma levels of CXCL16 in long-term smokers with normal LF and GOLD 1 patients suggest that soluble CXCL16 might be a new peripheral biomarker of COPD development risk in pre-COPD subjects. Furthermore, given that CXCL16 attracts (soluble form) and arrests (transmembrane form) CXCR6^+^ leukocytes or CXCR6^+^ leukocyte-platelet aggregates, our findings may support further research on CXCL16/CXCR6 axis as a potential therapeutic target to prevent lung immune cell infiltration and subsequent COPD development and progression.

## Data Availability

The raw data supporting the conclusions of this article will be made available by the authors, without undue reservation.
