# Supplementary material for: CXCL16/CXCR6 axis arises as a potential peripheral biomarker of early COPD development – results from a pilot study
Source: Front Med (Lausanne). 2025 Jul 9;12:1636360. doi: 10.3389/fmed.2025.1636360 (PMC12283661; doi:10.3389/fmed.2025.1636360)
Supplement: Supplementary file 1 [file Data_Sheet_1.pdf]

Supplementary Material

CXCL16/CXCR6 axis arises as a potential peripheral biomarker of early COPD development – Results from a pilot study

1 Determination of CXCR6 expression on platelets and leukocyte subsets by flow cytometry

A portion of heparinized blood was incubated with EDTA (10 mM, for 15 min at 37ºC) to promote platelet dissociation [1]. A total of 50 µL of heparinized blood was used for leukocyte-platelet aggregate immunophenotyping, 50 µL of EDTA-treated blood for platelet-free leukocyte immunophenotyping, and 20 µL of citrated blood for platelet immunophenotyping. Saturated amounts of fluorochrome-conjugated monoclonal antibodies (mAbs) were added to the blood samples (described below), along with 50 µL of brilliant staining buffer (BD Biosciences, San Jose, CA). Each sample was gently vortexed and incubated for 30 min at room temperature in the dark. Subsequently, 1× lysis buffer (BD Phosflow™ Lyse/Fix Buffer 5× concentrate, BD Biosciences) was added to each tube to lyse the erythrocytes. All samples were then run on a BD LSRFortessa™ X-20 flow cytometer (BD Biosciences) and data were analyzed using FlowJo® v10.0.7 software (FlowJo LLC, Ashland, OR). Results were expressed as the percentage of CXCR6^{+} platelets, leukocyte-platelet aggregates, or platelet-free leukocytes.

To determine CXCR6 platelet expression (CD41+ population, **Figure S1**, blood samples were incubated with a PerCP-Cy™5.5-conjugated mAb against human CD41 (1.0 µL, clone HIP8, IgG1) and a BV421-conjugated mAb against human CXCR6 (1.0 µL, clone 13B 1E5, IgG2A) (both from BD Biosciences).

Similarly, to determine CXCR6 expression on leukocyte subsets, blood samples were incubated with a BV421-conjugated mAb against human CXCR6 (2.5 µL, clone 13B 1E5, IgG2A; BD Biosciences) and several other fluorochrome-conjugated antibodies against human-specific surface markers, according to the different leukocyte subsets studied:

An FITC-conjugated mAb against CD16 (5 µL, clone 3G8, IgG1, BD Biosciences) was used to detect **neutrophils** (CD16^{+}) or **eosinophils** (CD16^{-}) (**Figure S2**).

A BV650-conjugated mAb against CD14 (1.25 µL, clone M5E2, IgG2A), an FITC-conjugated mAb against CD16 (5 µL, clone 3G8, IgG1) (both from BD Biosciences) and a BV510-conjugated mAb against CCR2 (2.5 µL, clone K036C2, IgG2A, BioLegend, San Diego, CA) were used to detect **total monocytes** (CD14^{+}), **classical monocytes** (Mon1, CD14^{++}CD16^{-}CCR2^{+}), **intermediate monocytes** (Mon2, CD14^{++}CD16^{+}CCR2^{+}), or **nonclassical monocytes** (Mon3, CD14^{+}CD16^{+}CCR2^{-}) (**Table S1**, **Figure S3**).

An APC-H7-conjugated mAb against CD3 (1.25 µL, clone SK7, IgG1), a BUV395-conjugated mAb against CD4 (1.25 µL, clone RPA-T4, IgG1), an FITC-conjugated mAb against CD8 (10 µL, clone RPA-T8, IgG1), an APC-conjugated mAb against CXCR3 (10 µL, clone 1C6/CXCR3, IgG1), a PE/Cy7-conjugated mAb against CCR6 (2.5 µL, clone 11A9, IgG1), an APC-conjugated mAb against CD25 (10 µL, clone M-A251, IgG1) and a BV650-conjugated mAb against CD127 (2.5 µL, clone HIL-

7R-M21, IgG1) (all from BD Biosciences) were used to detect **T-lymphocytes** ($CD3^{+}$), **cytotoxic T-cells** ($CD3^{+}CD8^{+}$), **T-helper cells** (Th, $CD3^{+}CD4^{+}$), **Th1 cells** ($CD4^{+}CXCR3^{+}CCR6^{-}$), **Th2 cells** ($CD4^{+}CXCR3^{-}CCR6^{-}$), **Th17 cells** ($CD4^{+}CXCR3^{-}CCR6^{+}$), or **regulatory T-cells** (Tregs; $CD4^{+}CD25^{+}CD127^{low}$) (**Table S2**, **Figures S4 and S5**).

An FITC-conjugated mAb against CD19 (2.5 µL, clone SJ25C1, IgG1, BioLegend) was used to detect **B-lymphocytes** ($CD19^{+}$, **Figure S6**).

For all leukocyte subsets, platelet-leukocyte aggregates ($CD41^{+}$) and platelet-free leukocytes ($CD41^{-}$) were detected using a PerCP-Cy™5.5-conjugated mAb against human CD41 (2.5 µL, clone HIP8, IgG1; BD Biosciences).

2 References

1. Marques, P.; Domingo, E.; Rubio, A.; Martinez-Hervás, S.; Ascaso, J.F.; Piqueras, L.; et al. Beneficial effects of PCSK9 inhibition with alirocumab in familial hypercholesterolemia involve modulation of new immune players. *Biomed Pharmacother*. 2022;**145**:112460. 10.1016/j.biopha.2021.112460

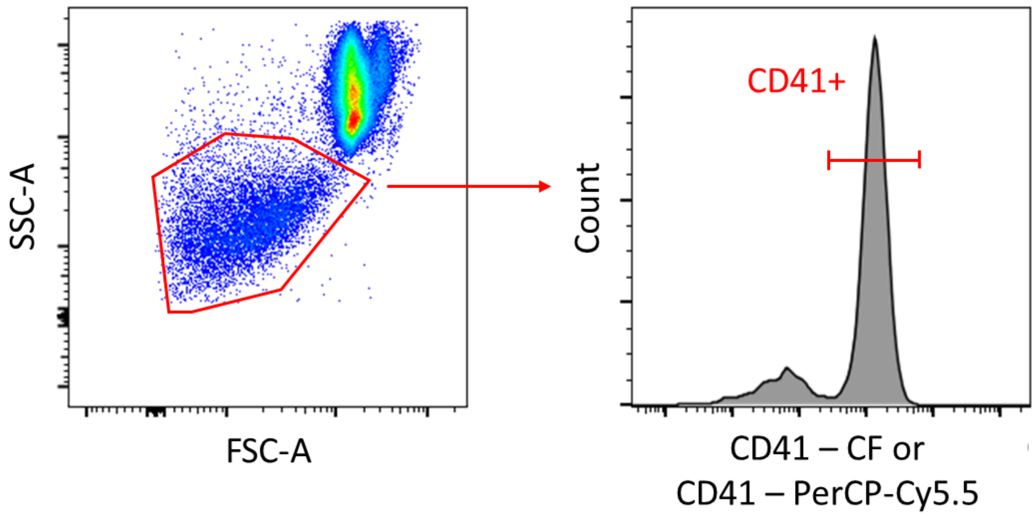

Figure S1. **Gating strategy for human platelets in whole blood according to morphological properties and CD41 detection by flow cytometry.** Platelets were gated according to a low side scatter (SSC-A) and forward scatter (FSC-A) in a logarithmic scale and defined as $CD41^+$ population.

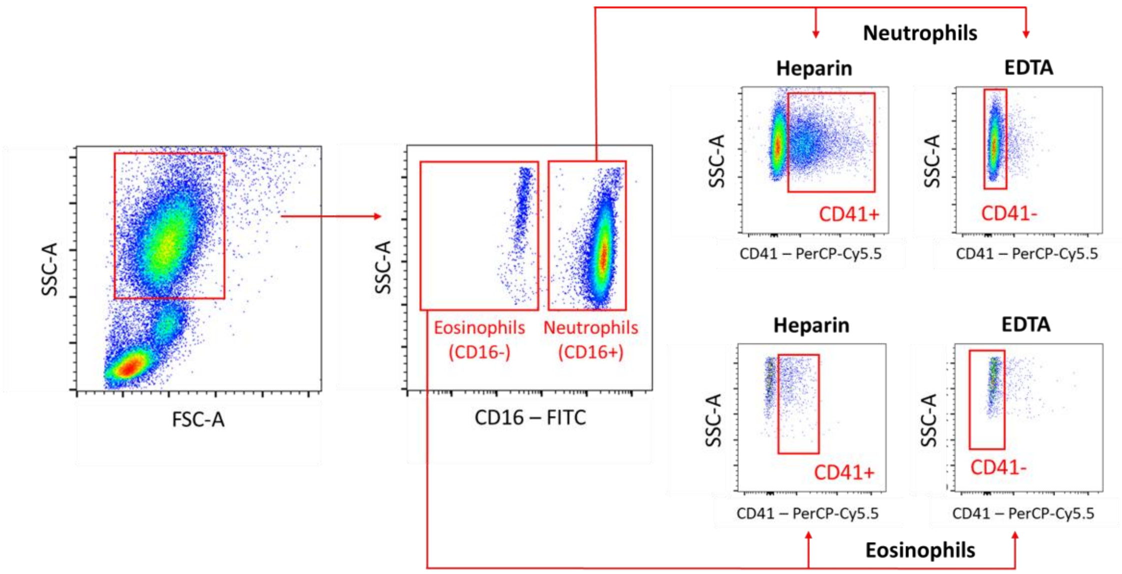

Figure S2. Gating strategy for human neutrophils and eosinophils in whole blood according to morphological properties and CD16 expression by flow cytometry. Populations were selected by morphology (high SSC-A). A CD16 antibody was used to detect neutrophils ($CD16^{+}$) and eosinophils ($CD16^{-}$). In heparinized blood, neutrophil-platelet-aggregates were selected as a $CD16^{+}CD41^{+}$ population and eosinophil-platelet aggregates as a $CD16^{-}CD41^{+}$ population; whereas platelet-free neutrophils were gated as a $CD16^{+}CD41^{-}$ population and platelet-free eosinophils as a $CD16^{-}CD41^{-}$ population from blood incubated with EDTA.

Table S1. Differential markers of monocyte subpopulations

Marker	Cellular population
**$CD14^{++}CD16^{-}CCR2^{+}$**	Monocyte type 1 (Mon1)
**$CD14^{++}CD16^{+}CCR2^{+}$**	Monocyte type 2 (Mon2)
**$CD14^{+}CD16^{+}CCR2^{-}$**	Monocyte type 3 (Mon3)

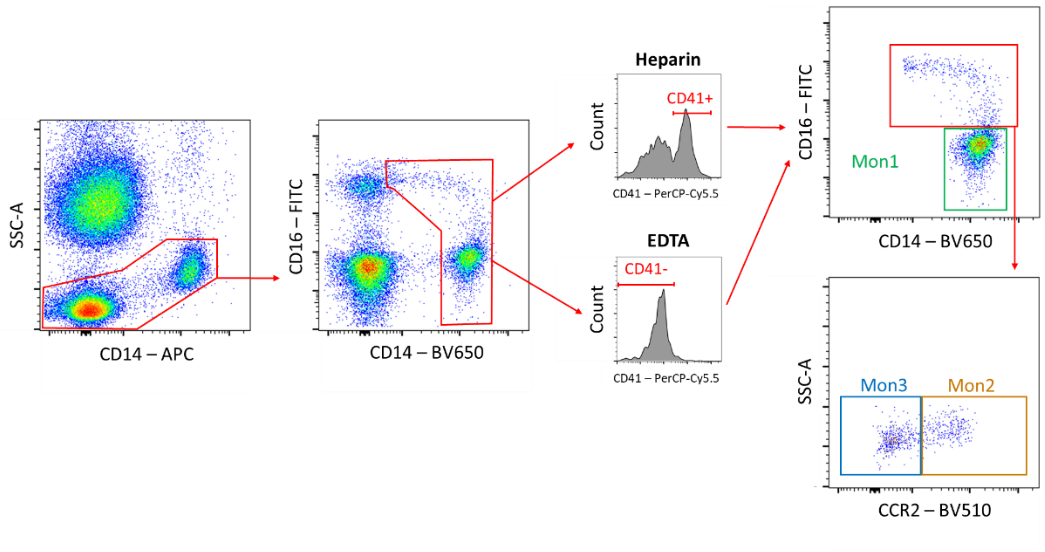

Figure S3. Gating strategy for human monocyte detection in whole blood by flow cytometry. Monocytes were selected by CD14 labelling and morphology (medium SSC-A). For the detection of monocyte subpopulations, CD16 and CCR2 markers were used. Monocyte-platelet complexes were selected as $CD14^{+}CD41^{+}$ populations in heparinized whole blood, and platelet-free monocytes were gated as $CD14^{+}CD41^{-}$ populations from blood incubated with EDTA.

Table 2. Differential markers of T-helper subpopulations

Marker	Cellular population
**CD4$^+$CXCR3$^+$CCR6$^-$**	T helper 1 (Th1)
**CD4$^+$CXCR3$^-$CCR6$^-$**	T helper 2 (Th2)
**CD4$^+$CXCR3$^-$CCR6$^+$**	T helper 17 (Th17)

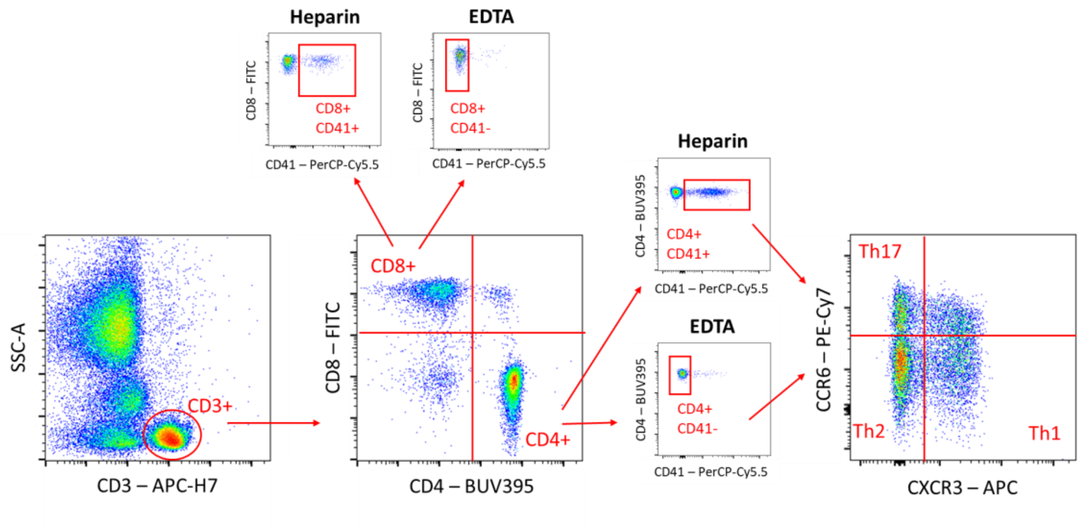

Figure S4. Gating strategy for human T-lymphocyte detection in whole blood by flow cytometry. T-lymphocytes were selected as a CD3$^+$ population and with a low SSC-A. Cytotoxic lymphocytes were selected as CD3$^+$CD8$^+$. In heparinized blood, cytotoxic lymphocyte-platelet complexes were selected as the CD3$^+$CD8$^+$CD41$^+$ population, whereas platelet-free cytotoxic lymphocytes were gated as CD3$^+$CD8$^+$CD41$^-$ from blood incubated with EDTA. T-helper (Th) lymphocytes were selected as the CD3$^+$CD4$^+$ population. In heparinized blood, Th lymphocyte-platelet complexes were selected as the CD3$^+$CD4$^+$CD41$^+$ population, whereas platelet-free Th lymphocytes were gated as a CD3$^+$CD4$^+$CD41$^-$ population from blood incubated with EDTA. Th lymphocyte subpopulations were detected with the markers CXCR3 and CCR6. In heparinized blood, Th1 lymphocyte-platelet complexes were selected as CD4$^+$CXCR3$^+$CCR6$^-$CD41$^+$, Th2 lymphocyte-platelet complexes were selected as CD4$^+$CXCR3$^-$CCR6$^-$CD41$^+$ and Th17 lymphocyte-platelet complexes were selected as CD4$^+$CXCR3$^-$CCR6$^+$CD41$^+$; whereas platelet-free Th lymphocyte subpopulations were gated as a CD4$^+$CXCR3$^+$CCR6$^-$CD41$^-$ (Th1), a CD4$^+$CXCR3$^-$CCR6$^-$CD41$^-$ (Th2) or a CD4$^+$CXCR3$^-$CCR6$^+$CD41$^-$ (Th17) population from blood incubated with EDTA.

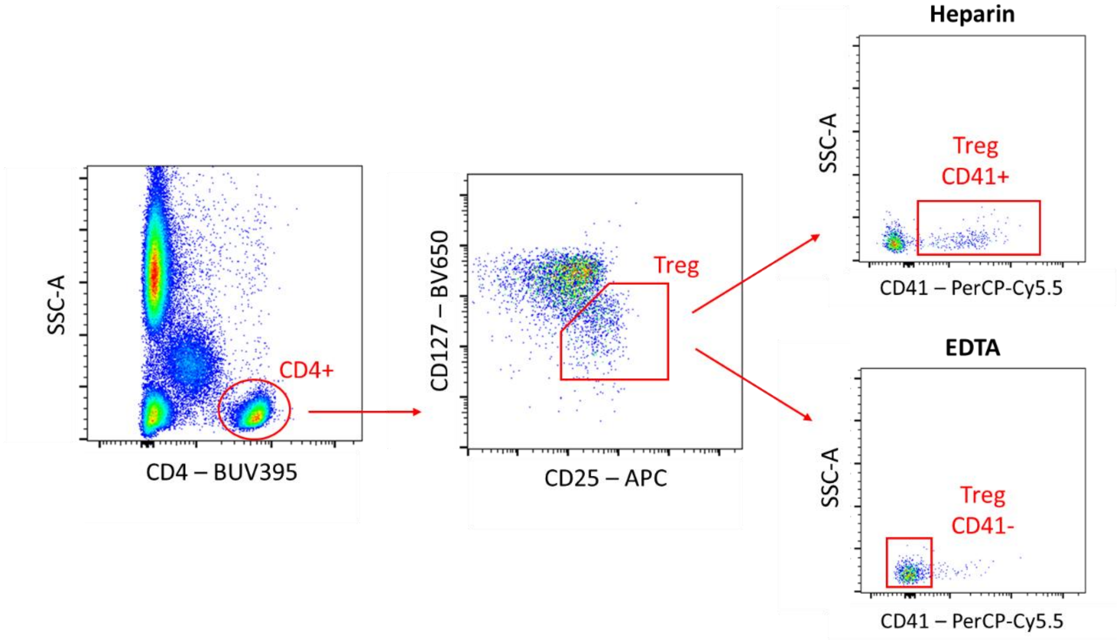

Figure S5. Gating strategy for human regulatory T-lymphocyte (Treg) detection in whole blood by flow cytometry. Treg lymphocytes were selected as the $CD4^{+}$ population and with a low SSC-A. Treg lymphocytes were detected with the markers CD127 and CD25. Treg lymphocyte-platelet complexes were selected as the $CD4^{+}CD127^{low}CD25^{+}CD41^{+}$ population from heparinized whole blood, whereas platelet-free Treg lymphocytes were gated as a $CD4^{+}CD127^{low}CD25^{+}CD41^{-}$ population from blood incubated with EDTA.

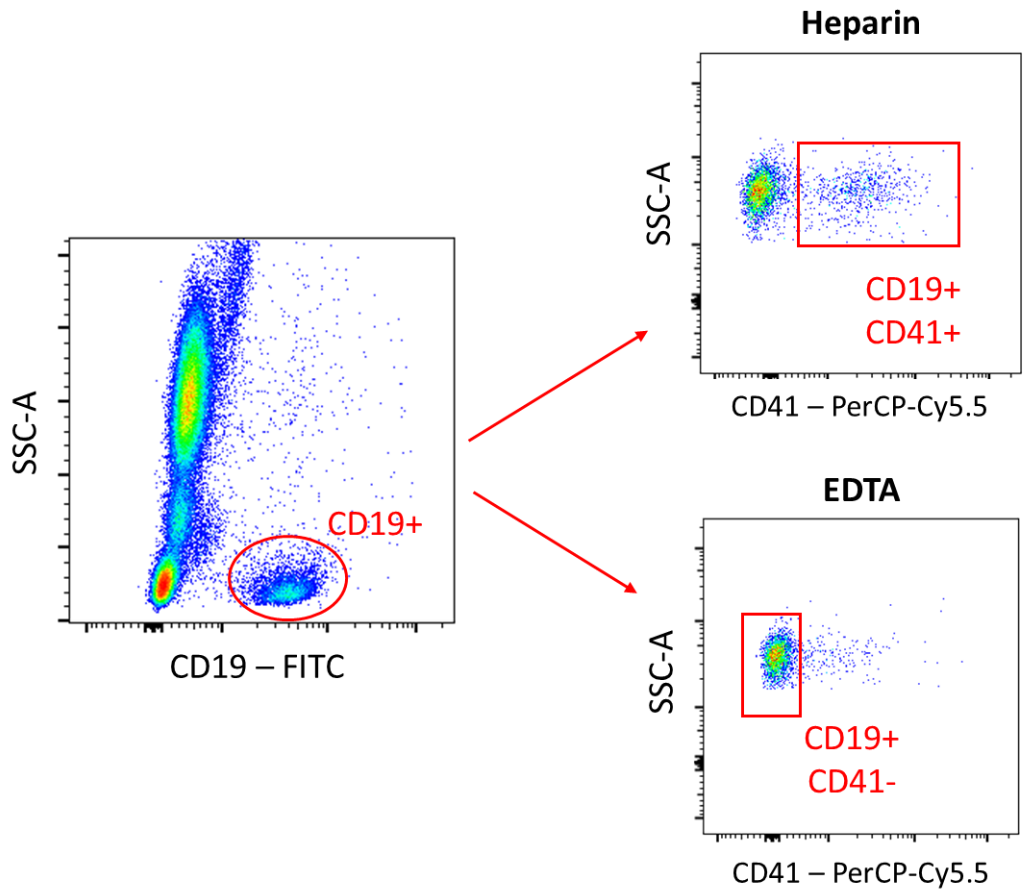

Figure S6. **Gating strategy for human B-lymphocyte detection in whole blood by flow cytometry.** B-lymphocytes were selected as a $CD19^{+}$ population and with a low SSC-A. In heparinized blood, B-lymphocyte-platelet complexes were selected as the $CD19^{+}CD41^{+}$ population, whereas platelet-free B-lymphocytes were gated as $CD19^{+}CD41^{-}$ from blood incubated with EDTA.

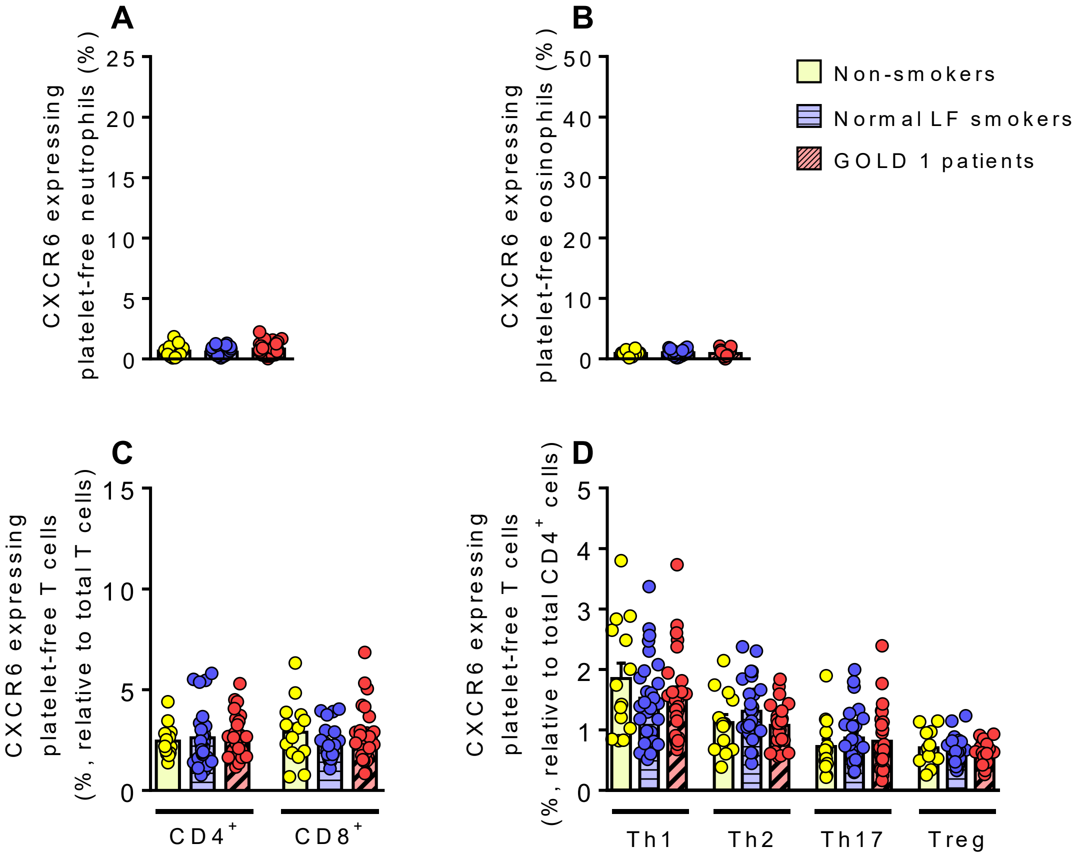

Figure S7. CXCR6 expression is negligible in platelet-free granulocytes and no differences were observed in platelet-free T cells between groups. Flow cytometry analysis of CXCR6 expression in platelet-free neutrophils ($CD16^{+}CD41^{-}$, **A**), eosinophils ($CD16^{-}CD41^{-}$, **B**) or T cells ($CD4^{+}CD41^{-}$ or $CD8^{+}CD41^{-}$, **C**). The same analysis was done for the different T helper cell subsets (Th1: $CXCR3^{+}CCR6^{-}$; Th2: $CXCR3^{-}CCR6^{-}$; Th17: $CXCR3^{-}CCR6^{+}$) and regulatory T cells (Treg: $CD25^{+}CD127^{low}$) free of platelets ($CD41^{-}$, **D**). Results are presented as the percentage of positive ($CXCR6^{+}$) cells. Values are expressed as mean ± SEM.
